# A cross-sectional study of the prevalence and correlates of post-traumatic stress among people attending acceptance and commitment therapy for chronic pain

**DOI:** 10.1177/20494637261497966

**Published:** 2026-10-16

**Authors:** Kezia Kadri, Cara Keating, Madeleine Anderson, David Novelli, Emily McBride, Whitney Scott

**Affiliations:** 1 INPUT Pain Management Unit, 8945 Guy’s & St Thomas’ NHS Foundation Trust, London, UK; 2 Health Psychology Section, Institute of Psychiatry, Psychology, and Neuroscience, 4616 King’s College London, London, UK; 3 Bariatric Psychology Service, Lewisham and Greenwich NHS Trust, London, UK

**Keywords:** trauma, post-traumatic stress, persistent pain, psychological flexibility, acceptance and commitment therapy

## Abstract

**Objective:**

Experiences of trauma are common among people with chronic pain, although estimates of comorbid post-traumatic stress vary widely across contexts. Acceptance and commitment therapy (ACT), which targets psychological flexibility to improve pain-related functioning, is increasingly delivered for people with pain. To inform treatment delivery, understanding of the prevalence and correlates of post-traumatic stress among those attending ACT-based treatment for pain is needed. This study aimed to identify the prevalence of post-traumatic stress and its associations with key pain-related outcomes and other relevant psychosocial factors at the start of an ACT-based treatment.

**Methods:**

This was a cross-sectional study using data collected within routine clinical care at a speciality pain management service in the UK. Four hundred and fifty-one participants completed standardized self-report questionnaires of post-traumatic stress symptoms (PTSS), pain-related outcomes (pain intensity and interference, work and social adjustment, and depression symptoms), psychological flexibility/inflexibility, and stigma at the start of an interdisciplinary ACT-based treatment for chronic pain.

**Results:**

Forty-seven percent of patients (*n* = 213) reported a traumatic exposure and 30% of the sample reported clinically meaningful PTSS. Greater severity of PTSS and probable post-traumatic stress disorder (PTSD) were associated with significantly worse pain-related outcomes, psychological flexibility/inflexibility, and stigma in bivariate analyses. PTSS severity and probable PTSD showed significant unique associations with poorer pain outcomes in regression models controlling for covariates and psychological flexibility/inflexibility.

**Conclusions:**

Post-traumatic stress is common among people engaging in ACT-based treatment for pain and associated with adverse pain outcomes. Trauma-informed practice is therefore important when providing ACT-based treatment for pain.

## Introduction

Chronic pain is often comorbid with mental health problems, particularly depression and anxiety.^1^ There is also recognition of the relevance of trauma among people with chronic pain.^2^ For some, exposure to extremely threatening experiences leads to post-traumatic stress disorder (PTSD), characterized by re-living, avoidance of reminders of the trauma, altered arousal/reactivity, and negative cognitions and mood.^3^

One meta-analysis found a 10% PTSD point prevalence in people with chronic pain,^4^ in contrast to a 3.5% 12-month prevalence in the general population.^5^ Similarly, a large proportion of those with PTSD experience chronic pain, with particularly high rates for veterans (around 50%) and refugees (20–80%).^6,7^ Importantly, post-traumatic stress is associated with increased pain severity, pain-related disability, and depression.^4,8–10^ Moreover, chronic pain and post-traumatic stress are, individually, associated with stigma,^11,12^ but research is needed to understand whether comorbid post-traumatic stress is associated with greater stigma among people with pain.

Taken together, many people seeking treatment for pain present with post-traumatic stress.^13,14^ Multidisciplinary pain treatments are now widely based on cognitive-behavioural principles.^15,16^ However, limited research has investigated the prevalence and correlates of post-traumatic stress among people engaging in these treatments and post-traumatic stress may be particularly relevant for those presenting to pain services. A Swedish study of patients assessed at a pain rehabilitation unit found that 72% reported a traumatic exposure, and 29% met diagnostic criteria for current PTSD.^8^ Research is needed to investigate the generalizability of these findings to patients seeking cognitive-behavioural pain treatment in other settings and among socio-demographically diverse groups. This can identify the degree to which trauma-informed and trauma-focused treatments are needed as part of, or in addition to, existing evidence-based cognitive-behavioural pain treatments.

Shared vulnerability and mutual maintenance models have been proposed to understand the pain-trauma link.^17^ Within these, people with increased sensitivity to threat-related stimuli and a tendency to focus on pain- or trauma-related cues are more likely to interpret bodily sensations (like pain or arousal) as dangerous. This contributes to fear, hypervigilance, and avoidance that maintain PTSD and chronic pain.^17^

More recent research highlights the psychological flexibility model to understand the pain-PTSD link.^18^ Psychological flexibility is the ability to persist with or change behaviour depending on one’s goals and values, whilst remaining open to and aware of difficult experiences in the present.^19^ This involves six processes: acceptance (willingness to have difficult experiences), cognitive defusion (separating thoughts from behaviour), present-moment awareness (focusing on the now), self-as-context (noticing one is separate from internal experiences), values (identifying what is important), and committed action (acting in line with values).^19^ Psychological flexibility is associated with improved pain outcomes.^20^ One study in a sample attending traditional cognitive-behavioural treatment for pain found that those with probable PTSD had lower psychological flexibility than those who did not, as well as higher depression, pain interference and severity.^18^ Pain acceptance, committed action, and cognitive fusion also mediated between trauma symptoms and pain interference.^18^ However, there is limited understanding of how all six psychological flexibility facets together relate to post-traumatic stress and pain-related outcomes.

The links between psychological flexibility, post-traumatic stress, and pain are relevant given that pain management treatments focused on psychological flexibility are increasingly implemented. Acceptance and commitment therapy (ACT) is a third-wave cognitive-behavioural intervention that targets psychological flexibility to improve quality of life with pain, with growing evidence.^21,22^ However, existing trauma-pain research has largely been conducted in the context of traditional cognitive-behavioural therapy. As such, there is a need to understand trauma prevalence, and how post-traumatic stress relates to pain outcomes and psychological flexibility for those engaging in ACT.

This study investigated the prevalence and correlates of post-traumatic stress among people attending ACT-based treatment for pain. We hypothesized that those with probable PTSD would report poorer pain outcomes and less psychological flexibility than those with subthreshold PTSS or no trauma exposure. The association between post-traumatic stress and pain-related outcomes controlling for demographic factors and psychological flexibility/inflexibility was also investigated. An exploratory analysis of the bivariate relationship between stigma and post-traumatic stress was also undertaken.

## Methods

### Design

A cross-sectional observational study was conducted using routine clinical data collected between January 2023 and April 2025. The study had ethical approval (South Central Oxford NHS Research Committee, 22/SC/0388).

### Participants

Participants were adults with chronic pain attending a pain management program within the UK’s National Health Service (NHS). Participants were eligible for the program if they were ≥18 years old and had pain ≥3 months that was associated with significant distress and/or disability. Participants were willing to complete an English-language group treatment focused on improving quality of life in the presence of pain rather than pain reduction. Those undergoing medical investigations, unable to move independently, or with significant psychological complexities judged as likely to interfere with treatment (e.g. active psychosis, active suicidal ideation, and substance abuse) were excluded. Eligibility was assessed by a physiotherapist and psychologist/occupational therapist. All patients were invited to complete standardized questionnaires as part of routine care. Patients who consented to their data being used for research were included in this study.

### Treatment context

Although this study presents baseline data only, brief details of the treatment are provided for context. The treatment is a three-week residential (four days per week) ACT-based^21^ multidisciplinary pain management program. The program involves group-delivered psychology, physiotherapy, occupational therapy, and nursing sessions. The program aims to improve quality of life with pain by targeting psychological flexibility.^20^ Experiential exercises, metaphors, mindful movement, and values-based goal-setting are used to this end.

### Measures

Participants completed standardized self-report measures at the start of treatment.

#### Clinical and demographic information

Questions asked about pain history (location/duration) and demographic information (age, gender, ethnicity, education, and work). Burden of comorbid physical and mental health conditions was captured using the Multimorbidity Treatment Burden Questionnaire (MTBQ) for descriptive purposes.^23^

#### Trauma exposure and post-traumatic stress symptom severity

The PTSD Checklist-5 (PCL-5)^24^ was used to measure post-traumatic stress symptoms. Trauma exposure was assessed using an adapted PCL-5 criterion A assessment. As self-report measures were completed without clinical support, the clinical team judged it inappropriate for patients to recount extensive details of their trauma histories. Therefore, instead of asking participants to identify/describe the very stressful experience, the adapted version involved one yes/no question asking, ‘*Did your stressful experience(s) involve actual or threatened death, serious injury, or sexual violence?*’ Those responding ‘Yes’ completed the PCL-5. The rest of the Criterion A assessment was unchanged.

The PCL-5 has 20-items assessing the Diagnostic and Statistical Manual of Mental Illnesses (DSM-5)^3^ symptoms of PTSD over the past month. Items are rated from 0 (not at all) to 4 (extremely), with a total score ≥31 indicating probable PTSD.^24^ Probable PTSD can also be determined by symptom cluster: cluster B (intrusive symptoms: items 1–5), C (avoidance: items 6–7), D (negative cognition/mood: items 8–14), and E (altered arousal/reactivity: items 15–20). Those scoring ≥2 for one B item, one C item, two D and two E items met criteria for probable PTSD.^24^ The PCL-5 is a well-validated and internally reliable measure.^25^

#### Key pain-related outcome variables

Based on the IMMPACT recommendations for core chronic pain outcomes,^26^ the following variables were measured:

##### Pain intensity

A single item rating scale was used to indicate average pain intensity in the last week, ranging from 0 (no pain) to 10 (pain as bad as you can imagine). This item has been shown to have good validity and reliability for assessing pain intensity.^27^

##### Pain interference

Pain interference was measured using the Brief Pain Inventory – Interference Scale (BPI-I).^28^ This consisted of 7 items asking about how much, over the past week, pain interfered with daily life, including mood, life enjoyment, sleep, general activity, work, relationships, and walking. Items were assessed from 0 (does not interfere) to 10 (completely interferes). The average of the items was used, with higher scores indicating greater interference. The BPI-I is widely used and validated among people with chronic pain.^29^

##### Impact on functioning

The impact of pain on functioning was further assessed using the Work and Social Adjustment Scale (WSAS).^30^ The five items assess the impact of health conditions on work, home, relationships, and social activities. Items were rated from 0 (not at all) to 8 (very severely). Higher total scores indicate greater functional impairment. The WSAS has high internal reliability and has been validated in people with chronic pain.^31^

##### Depression

The Patient Health Questionnaire-9 (PHQ-9)^32^ was used to measure depression symptoms with nine items, including low mood, sleep disturbance and concentration difficulties, over the past 2 weeks. Items were rated from 0 (not at all) to 3 (nearly every day), with higher total scores indicating more severe depression symptoms. The PHQ-9 has shown reliability and validity as a measure of depressive symptoms across chronic pain samples.^32^

#### Other psychosocial variables

##### Psychological flexibility and inflexibility

The Multidimensional Psychological Flexibility Inventory (MPFI)^33^ is a 60-item measure assessing each process of psychological flexibility and inflexibility (i.e. acceptance/experiential avoidance, present-moment awareness/lack of present-moment awareness, self-as-context/self-as-content, cognitive defusion/fusion, contact with values/lack of contact with values, committed action/inaction). Items are rated with respect to the previous 2 weeks, from 1 (never true) to 6 (always true). Despite being strongly related, psychological flexibility and psychological inflexibility are partially distinct and not simply the inverse of one another.^34^ Flexibility emphasizes adaptive and values-oriented behaviour, whereas inflexibility captures more rigid and avoidant behaviour.^35^ Average scores for each individual subscale were used for bivariate correlations. The overall flexibility and inflexibility subscales were used for all other analyses to reduce the number of variables in the models. Higher average scores indicate greater levels of flexibility or inflexibility. The MPFI has shown strong reliability and validity in people with pain.^34^

##### Stigma

Stigma was measured given previous research showing its relevance for people with pain^11^ and, separately, for people with PTSD.^12^ Stigma was therefore included for exploratory purposes to examine its bivariate associations with post-traumatic stress in our sample with chronic pain.^11^ The eight-item Stigma Scale for Chronic Illness (SSCI-8)^36^ was used to measure internalized and enacted stigma in relation to one’s illness. Items were rated from 1 (never) to 5 (always), with higher total scores indicating higher stigma. The SSCI-8 is internally consistent and valid for use with people with chronic pain.^37^

### Procedure

To minimize bias, patients attending the program were consecutively sampled. All patients starting treatment were invited to complete standardized and validated questionnaires online before the first day of treatment, or a paper-copy on the first day if they could not complete online. Participants provided consent for their data to be used for research.

### Statistical analysis

Mean imputation was used for participants with ≤10% missing data on individual questionnaires. To calculate a valid psychological flexibility/inflexibility score, participants needed a score for all individual subscales. Trauma groups were categorized using the PCL-5 total.^25^ Previous research found no differences in pain outcomes for those without trauma exposure and those with trauma exposure and subthreshold PTSS^8^; therefore, the groups were collapsed into a ‘no trauma exposure/subthreshold PTSS’ group and a ‘probable PTSD’ group. This allowed comparison between patients with and without clinically meaningful PTSS.^18^ Probable PTSD was also calculated using the cluster-based criteria.^24^ When calculating cluster-based scores for the PCL-5, missing data were not imputed, given that the specific item value was key to meeting the criteria.

Chi-square tests were used to examine group differences for categorical variables. Categorical variables were dichotomized to ensure sufficient numbers per group: gender (male/female), ethnicity (ethnically minoritized/white), working status (employed including part time and student/unemployed or retired). One-way ANOVAs were conducted for parametric continuous variables between groups. Kruskal–Wallis tests were used for non-parametric data, and Welch tests reported where distributions were non-homogenous.

Among participants who reported a traumatic exposure and completed the PCL-5, bivariate Pearson’s correlations were conducted to examine associations between variables. Hierarchical multiple linear regressions were then used to examine the association between PTSS and pain-related outcomes (pain intensity and interference, work and social adjustment, depression), controlling for gender, ethnicity, and age, based on prior research.^38^ Individual models controlled for additional demographic variables that showed significant associations with the dependent variable or PCL-5 scores. Pain intensity was included in the second block of each model (except the model predicting pain intensity). Separate models were conducted for psychological flexibility and inflexibility, given potential multicollinearity.^34^ Stigma was only included as an exploratory variable for bivariate analyses and was not the main focus of the study. As such, it was not included in regressions to keep the models more parsimonious given the range of variables already included. Sensitivity analyses using trauma as a categorical variable (i.e. no exposure/subthreshold PTSS vs probable PTSD) were conducted using the same outcome variables and covariates.

Normality assumptions for multiple regressions were assessed using histograms, q-q plots and partial regression plots. Where assumptions were not met, standardized residuals with values │2.5│ were identified as potential outliers, and models rerun with these excluded. If exclusion of outliers did not alter model findings or normality, residuals were retained and the original model reported. Analyses were conducted on SPSS (Version 27). The sample size was determined pragmatically based on the number of participants recruited within the specified dates and was deemed sufficient given previous similar research.^8,18^

## Results

Five hundred and three participants completed questionnaires. Of these, 451 (90%) consented for their data to be used for research. Sample characteristics are in Table 1.

**Table 1.** Sample characteristics.

Variable	Mean (SD) or frequency (%)
Age^a^	50.25 (13.41)
Gender	
Female	366 (81.2)
Male	76 (16.9)
Prefer not to say	7 (1.6)
Missing	2 (0.4)
Ethnicity	
Asian	29 (6.4)
Black	77 (17.1)
Mixed	33 (7.3)
Other	17 (3.8)
White	294 (65.2)
Missing	1 (0.2)
Pain duration in years^b^	14.39 (13.17)
Pain all over body	
Yes	315 (69.8)
No	126 (27.9)
Missing	10 (2.2)
Main pain location	
Head, face or mouth	12 (2.7)
Neck region	15 (3.3)
Upper shoulder or upper limbs	38 (8.4)
Chest region	5 (1.1)
Abdominal region	10 (2.2)
Lower back, lumbar spine, sacrum, and coccyx	213 (47.2)
Lower limbs	72 (16)
Pelvic region	18 (4)
Anal or genital region	2 (.4)
Missing	66 (14.6)
Comorbid physical health condition	
Yes	374 (82.9)
No	69 (15.3)
Missing	8 (1.8)
Comorbid mental health condition	
Yes	251 (55.7)
No	190 (42.1)
Missing	10 (2.2)
Health effort^c^	27.03 (9.59)
Working status	
Employed	191 (42.4)
Unemployed	202 (44.8)
Student	7 (1.5)
Retired	44 (9.8)
Missing	7 (1.5)
Traumatic exposure	
Yes	213 (47.2)
No	218 (48.3)
Missing	20 (4.4)
Years since traumatic exposure^d^	19.27 (15.58)
Experience of traumatic exposure^e^	
Directly	163 (78.4)
Witnessed	40 (20.1)
Learnt about happening to close family/friend	39 (19.9)
Repeatedly exposed to details via job	5 (2.6)
Other	7 (3.6)
Cause of death for traumatic exposures involving death^e^	
Accident/violence	36 (18.7)
Natural causes	35 (18.1)
Not applicable	122 (63.2)

*Note. N* = 451 unless otherwise specified. ^a^ = *N* = 409, ^b^ = *N* = 420, ^c^ = *N* = 442, ^d^ = *N* = 187, ^e^= *N* = 213 (those who completed the Criterion A assessment; multiple exposures could be selected), SD = standard deviation.

### Prevalence of post-traumatic stress

Two-hundred and thirteen participants (47%) reported a traumatic exposure. One participant who reported a traumatic exposure did not complete the PCL-5 so was excluded from further analysis. Therefore, 212 participants were included in PCL-5 analyses. A slightly larger proportion of participants were categorized as having probable PTSD using the total score (136/212 participants) versus the cluster-based criteria (125/212 participants). Approximately 85% of participants were categorized the same according to both criteria (65 participants met neither criteria, 114 participants met both). Twenty-two participants (10%) were categorized as having probable PTSD according to the total score but did not meet the cluster-based criteria, whereas 11 (5%) were not categorized as having probable PTSD according to the total score, but did meet the cluster-based criteria.

### Group comparison of demographic and clinical characteristics

Chi-squared tests found no significant differences between the two groups by gender, ethnicity, working status, or pain location. Average pain intensity was not significantly different between groups. All other variables were significantly higher for the probable PTSD group, except for psychological flexibility, which was significantly lower for this group (Table 2).

**Table 2.** Between-group comparisons for continuous self-report measures.

Mean (SD) (*n*)				Between-group test	Df	F/adjusted F	*p*-value	Kruskal–Wallis H	Adjusted *p*-value
Variable	Total sample	No trauma exposure/subthreshold PTSS	Probable PTSD						
Pain intensity	7.41 (1.53) 448	7.35 (1.51) 292	7.42 (1.51) 136	ANOVA	1	0.22	.639	​	​
Pain interference	7.57 (1.67) 448	7.38 (1.68) 292	7.94 (1.61) 136	ANOVA	1	10.78	.001	​	​
Impact on functioning	30.94 (7.19) 444	29.73 (7.57) 293	33.22 (5.82) 135	Kruskal–Wallis	1	​	​	22.54	<.001
Depression	16.80 (5.77) 441	15.53 (5.76) 292	19.74 (4.59) 134	Kruskal–Wallis	1	​	​	12.45	<.001
Psychological flexibility	3.28 (.91) 432	3.34 (.93) 279	3.08 (.86) 121	Kruskal–Wallis	1	​	​	49.77	<.001
Psychological inflexibility	3.44 (.89) 431	3.22 (.84) 279	3.89 (.82) 125	ANOVA, Welch	1	7.17	.008	​	​
Stigma	22.23 (6.89) 435	20.48 (6.48) 288	25.56 (6.35) 132	ANOVA	1	56.35	<.001	​	​

*Note.* SD = standard deviation, n = sample size, Df = degrees of freedom.

### Correlations among variables

Except for non-significant correlations with acceptance, present-moment awareness, and experiential avoidance, all variables were significantly associated with PCL-5 scores (Table 3). There were large correlations between PCL-5 scores and depression and psychological inflexibility. Correlations with psychological inflexibility subscales were larger than with psychological flexibility subscales.

**Table 3.** Correlations between PCL-5 total scores and other study variables.

Variable	PCL-5 total score
Pain intensity	.20*
Pain interference	.34**
Depression	.63**
Psychological flexibility	−.24*
Acceptance	.11
Present-moment awareness	.01
Self-as-context	−.21*
Defusion	−.27**
Values	−.29**
Committed action	−.32**
Psychological inflexibility	.62**
Experiential avoidance	.11
Lack of contact with present	.31**
Self-as-content	.51**
Fusion	.59**
Lack of contact with values	.47**
Inaction	.62**
Stigma	.43**

*Note.* * = statistically significant at *p* < .01, ** = statistically significant at *p* < .001. PCL-5 = PTSD Checklist for DSM-5.

### Multiple regressions

Multiple regression analyses examined whether PTSS severity was associated with pain-related outcomes after controlling for covariates. PTSS was a significant associated factor in all models. Controlling for covariates, PTSS accounted for an additional 4%, 5%, 6% and 44% of the variance in pain intensity, interference, impact on functioning, and depression, respectively. There were small significant effects in the models for pain intensity (*n* = 181, β = .21, *p* = .006), interference (*n* = 181, β = .23, *p* < .001) and impact on functioning (*n* = 180, β = .25, *p* < .001). PTSS was most strongly associated with depression (*n* = 179, β = .58, *p* < .001).

### Sensitivity analyses for associations between depression symptoms and PCL-5 symptom clusters

Due to item overlap between the PHQ-9 and PCL-5 Cluster D (negative cognitions and mood), sensitivity analyses were conducted for multiple regressions between PHQ-9 scores and each PCL-5 cluster (Supplemental Table 1). Each individual cluster was significantly associated with depression, controlling for demographics and pain intensity. Cluster D accounted for the most additional variance (β = .60, *p* < .001) compared to other clusters. However, all clusters showed medium to large effects (β = .41 to β = .60).

### Regression models including psychological flexibility and inflexibility

Additional models were conducted including psychological flexibility/inflexibility (Tables 4 and 5). Partial regression plots showed that linearity assumptions were not met for some models. Removal of standardized residuals did not alter assumptions or findings, so models with residuals are reported. Psychological *flexibility* accounted for 0% (pain intensity) to 13% (depression) of the variance in the models. Controlling for psychological flexibility, PCL-5 scores remained significantly associated with all the outcomes. Psychological *inflexibility* accounted for 0% (pain intensity) to 28% (depression) of the variance in the models. Controlling for psychological inflexibility, PCL-5 total scores remained significantly associated with all the outcomes.

**Table 4.** Regression models including psychological flexibility and post-traumatic stress symptom severity as factors associated with pain outcomes.

	Independent variable	F Change	Df	*p*	R^2^_adj_	ΔR^2^_adj_	β	Unstandardized B (SE)	*p*
Model 1 – Dependent: Pain intensity									
Step 1	Age	1.49	(4, 187)	.206	.01		.11	0.01	.130
	Gender						.01	0.02	.943
	Ethnicity						.02	0.05	.843
	Pain location						−.10	−0.10	.157
Step 2	Psychological flexibility	0.01	(1, 186)	.920	.01	.00	.04	0.04	.561
Step 3	PCL-5	6.40	(1, 185)	.012	.03	.02	.19	0.19	.012
Model 2 – Dependent: Pain interference									
Step 1	Age	1.12	(5, 186)	.352	.00		−.02	−0.00 (.01)	.729
	Gender						−.03	−0.13 (.27)	.637
	Ethnicity						−.03	−0.09 (.21)	.682
	Pain location						−.04	−0.13 (.23)	.570
	Work status						.07	0.23 (.20)	.266
Step 2	Pain intensity	71.35	(1, 185)	<.001	.28	.28	.49	0.55 (.07)	<.001
Step 3	Psychological flexibility	9.15	(1, 184)	.003	.31	.03	−.13	−0.24 (.12)	.036
Step 4	PCL-5	10.47	(1, 183)	.001	.34	.03	.21	.02 (.01)	.001
Model 3 – dependent: Impact on functioning									
Step 1	Age	3.69	(5, 186)	.003	.07		.04	0.02 (.04)	.555
	Gender						−.02	−0.41 (1.23)	.739
	Ethnicity						−.06	−0.91 (.98)	.353
	Pain location						−.10	−1.62 (1.03)	.116
	Work status						.19	2.74 (.93)	.004
Step 2	Pain intensity	31.71	(1, 185)	<.001	.20	.13	.33	1.58 (.31)	<.001
Step 3	Psychological flexibility	6.46	(1, 184)	.012	.22	.02	−.10	−0.82 (.52)	.119
Step 4	PCL-5	12.73	(1, 183)	<.001	.27	.05	.24	0.09 (.03)	<.001
Model 4 – dependent: Depression									
Step 1	Age	3.33	(5, 186)	.007	.06		−.06	−0.03 (.02)	.249
	Gender						−.05	−0.73 (.82)	.377
	Ethnicity						−.06	−0.75 (.65)	.249
	Pain location						−.16	−2.03 (.69)	.004
	Work status						.05	0.59 (.62)	.347
Step 2	Pain intensity	11.46	(1, 185)	<.001	.11	.05	.14	0.55 (.21)	.008
Step 3	Psychological flexibility	33.00	(1, 184)	<.001	.24	.13	−.23	−1.47 (.35)	<.001
Step 4	PCL-5	86.98	(1, 183)	<.001	.48	.24	.53	0.16 (.02)	<.001

*Note.* All Standardized β were from final regression models. Gender, ethnicity, pain location, and work status were dichotomized. Reference categories for dichotomized variables were male (gender), white (ethnicity), pain location not all over (pain location), employed including part time and student (work status). Df = degrees of freedom, ΔR^2^adj = change in R^2^, SE = standard error. PCL-5 = PTSD Checklist for DSM-5.

**Table 5.** Regression models including psychological inflexibility and post-traumatic stress symptom severity as factors associated with pain outcomes.

	Independent variable	F Change	Df	*p*	R^2^_adj_	ΔR^2^_adj_	β	Unstandardized B (SE)	*p*
Model 1 – Dependent: Pain intensity									
Step 1	Age	1.49	(4, 187)	.206	.01		.11	0.01 (.01)	.158
	Gender						.01	0.04 (.29)	.881
	Ethnicity						.01	0.03 (.24)	.908
	Pain location						−.11	−0.36 (.24)	.146
Step 2	Psychological inflexibility	0.83	(1, 186)	.362	.01	.00	−.08	−0.13 (.16)	.407
Step 3	PCL-5	5.92	(1, 185)	.016	.04	.03	.23	0.02 (.01)	.016
Model 2 – Dependent: Pain interference									
Step 1	Age	1.12	(5, 186)	.352	.00		−.03	−0.00 (.01)	.646
	Gender						−.04	−0.19 (.27)	.489
	Ethnicity						−.03	−0.11 (.22	.609
	Pain location						−.03	−0.10 (.23)	.658
	Work status						.07	0.23 (.21)	.259
Step 2	Pain intensity	71.35	(1, 185)	<.001	.28	.28	.49	0.55 (.07)	<.001
Step 3	Psychological inflexibility	10.65	(1, 184)	.001	.34	.06	.09	0.16 (.15)	.287
Step 4	PCL-5	5.55	(1, 183)	.019	.36	.02	.19	0.02 (.01)	.019
Model 3 - dependent: Impact on functioning									
Step 1	Age	3.69	(5, 186)	.003	.07		.04	0.02 (.04)	.580
	Gender						−.03	−0.62 (1.23)	.613
	Ethnicity						−.06	−0.93 (.99)	.347
	Pain location						−.10	−1.52 (1.03)	.140
	Work status						.19	2.75 (.93)	.004
Step 2	Pain intensity	31.71	(1, 185)	<.001	.20	.13	.33	1.58 (.31)	<.001
Step 3	Psychological inflexibility	11.51	(1, 184)	<.001	.24	.04	.09	0.73 (.68)	.286
Step 4	PCL-5	6.34	(1, 183)	.013	.26	.02	.21	0.08 (.03)	.013
Model 4 – dependent: Depression									
Step 1	Age	3.33	(5, 186)	.007	.06		−.05	−0.02 (.02)	.344
	Gender						−.07	−1.14 (.82)	.166
	Ethnicity						−.05	−0.62 (.66)	.346
	Pain location						−.15	−1.85 (.69)	.008
	Work status						.05	0.60 (.62)	.346
Step 2	Pain intensity	11.46	(1, 185)	<.001	.11	.05	.15	0.56 (.21)	.007
Step 3	Psychological inflexibility	86.08	(1, 184)	<.001	.39	.28	.29	1.87 (.45)	<.001
Step 4	PCL-5	33.39	(1, 183)	<.001	.48	.09	.40	0.12 (.02)	<.001

*Note.* All Standardized β were from final regression models. Gender, ethnicity, pain location, and work status were dichotomized. Reference categories for dichotomized variables were male (gender), white (ethnicity), pain location not all over (pain location), employed including part time and student (work status). Df = degrees of freedom, ΔR^2^adj = change in R^2^, SE = standard error. PCL-5 = PTSD Checklist for DSM-5.

### Regressions with trauma as categorical variable

Sensitivity analyses using trauma as a categorical variable were conducted (Supplemental Tables 2-4). Normality assumptions were not met. However, removal of outliers did not change findings or normality deviations, so models with all residuals are presented. Probable PTSD was significantly associated with pain interference (*n* = 379, β = .16, *p* < .001), impact on functioning (*n* = 378, β = .20, *p* < .001), and depression (*n* = 377, β = .33, *p* < .001). Controlling for psychological flexibility or psychological inflexibility, probable PTSD remained significantly associated with all outcome variables, except for pain intensity.

## Discussion

In this study within a publicly funded healthcare system in the UK, nearly half of patients beginning intensive ACT-based treatment for pain reported a trauma exposure, and nearly two-thirds of those with a traumatic exposure had probable PTSD. Probable PTSD and more severe PTSS were associated with worse pain-related outcomes, psychological flexibility/inflexibility, and stigma. The results highlight the relevance of considering post-traumatic stress among people engaging in psychologically based treatment for pain.

Thirty percent of the baseline sample in the current study were categorized as having probable PTSD using the PCL-5 total score. This is substantially higher than the 12-month general population prevalence of 3.5%.^5^ It is also higher than the 10% point prevalence of PTSD in people with chronic pain reported in a previous meta-analysis.^4^ The prevalence using the cluster method in the current sample was slightly lower (28%). There has been wide variability in prevalence estimates of PTSD among people with pain, attributed to the specific diagnostic classifications and measures used.^4,39^ Estimates may also vary as a function of healthcare context and populations assessed, where greater prevalence may be observed in publicly funded systems that are more likely to serve marginalized communities at greater risk of post-traumatic stress.^40^

Probable PTSD in the current study was similar to a Swedish sample attending their first assessment within a speciality government-funded pain service (29%).^8^ The current data were collected *after* patients completed their first multidisciplinary assessment within the pain service. This is notable, as a previous audit within the service identified that nearly one third of patients are discharged following initial assessment.^41^ A frequent reason for discharge following initial assessment is the complexity of psychosocial factors, including post-traumatic stress, judged as likely to interfere with engagement in short-term and group-based ACT for pain.^41^ Therefore, the prevalence of probable PTSD among people presenting for initial assessment within the service is likely to be even higher.

Probable PTSD and PTSS severity were both associated with greater pain interference, functional impact, and depression controlling for covariates and psychological flexibility/inflexibility in regression analyses. This is consistent with research showing adverse associations between post-traumatic stress and pain-related outcomes.^8–10^ Despite this cross-sectional evidence, emerging research suggests that pre-treatment PTSD/PTSS may not be associated with worse outcomes following CBT or ACT for pain.^10,42^ Importantly, though, one study found that while approximately 30% of trauma-exposed participants experienced a clinically meaningful improvement in PTSS following a CBT-based pain program, 20% reported a meaningful exacerbation in these symptoms.^42^ Therefore, investigation of adverse effects of ACT-based pain management with respect to trauma symptoms is needed.

The association between PTSS and depression is worth considering. There was a strong bivariate correlation between these variables, consistent with previous findings.^8^ This may be partially due to overlap between depression and the ‘negative cognitions and mood cluster’ of PTSD. However, sensitivity analyses indicated that each PTSD symptom cluster was uniquely associated with depression. Thus, symptom overlap may not be the only factor accounting for this association. The association between PTSD and depression is complex, and numerous studies have investigated causal relationships and shared vulnerabilities.^43^ Traditional cognitive-behavioural approaches for pain have highlighted the role of variables such as pain-related worrying and pain self-efficacy in contributing to depression among people with pain.^44,45^ The current finding highlights the importance of targeting trauma-related distress to manage depression for some people with pain.

The psychological flexibility model has more recently been applied to understand the pain-PTSD link.^18^ PTSD/PTSS severity have been associated with lower pain acceptance and committed action, and higher cognitive fusion, but not values-based action.^18^ Although PTSS were not significantly associated with all individual MPFI subscales, there were significant correlations between PTSS scores and the comprehensive psychological flexibility/inflexibility scores in the current study. Interestingly, there was a large correlation between psychological inflexibility and PTSS and a small correlation between flexibility and PTSS. Using the MPFI, a previous study also reported stronger associations between psychological inflexibility versus flexibility and adverse pain-related outcomes.^34^ The MPFI inflexibility and PCL-5 items are all negatively framed. As such, the relative strength of their association may reflect a negative response bias.^34^ The findings further support psychological flexibility and inflexibility as related but partially distinct constructs.^33,34^

The inclusion of psychological flexibility/inflexibility reduced some of the variance in pain-related outcomes accounted for by PTSS/PTSD, although these remained significant associated factors. This is consistent with cross-sectional mediation analyses showing that facets of psychological flexibility/inflexibility mediate between PTSD and pain intensity and interference.^18^ Taken together, emerging evidence suggests that targeting psychological flexibility/inflexibility may have some benefits for post-traumatic stress and chronic pain outcomes. However, the cross-sectional nature of these studies precludes causal interpretation, and the reverse directions of effects are also plausible. Longitudinal and experimental intervention research are needed to directly test the causal nature of relationships between these variables.

The results highlight the importance of trauma-informed practice when delivering interdisciplinary pain care. Trauma-informed practice recognizes the widespread impacts of trauma and uses a strengths-based approach to support service engagement and reduce re-traumatization.^46^ It includes principles of safety, trustworthiness, choice, collaboration, empowerment, and cultural considerations.^46^ Ongoing reflection about how to deliver ACT-based pain management based on these principles is needed. The findings highlight the need for future research examining whether outcomes following ACT-based treatment for pain differ for those with clinically significant post-traumatic stress. Notably, people with the most complex PTSD presentations may struggle to access ACT-based pain management. Indeed, people with severe mental illness are often excluded from psychologically based pain treatments.^47^ Research is needed to understand how to meet the pain management needs of people with severe mental illness, including complex presentations of PTSD. Some patients may benefit from engaging with trauma-focused psychological therapy^48^ before or in addition to ACT-based pain management. Innovations in delivering pain care, such as up-skilling clinicians in mental health services in pain management, could also help address these disparities.^47^

Several limitations must be considered. Firstly, we did not conduct an in-depth structured clinical interview to diagnose PTSD. We also did not include an extended Criterion A assessment. We used a brief criterion A assessment, which was deemed appropriate with respect to trauma-informed practice, so that patients were not asked to provide extensive details about their trauma in the absence of appropriate clinical support. However, this may have influenced the estimated prevalence of probable PTSD. Approximately 4% of participants did not complete the traumatic exposure question. Additionally, some participants who did not complete the PCL-5 at baseline did complete this at post-treatment (data not reported here). Therefore, some individuals may not have felt able to reflect on these experiences at pre-treatment, which may have contributed to an underestimation of the baseline prevalence.

There were a relatively smaller number of participants (*n* = 191) in the analyses examining associations between PTSS severity and pain-related outcomes. However, the ratio of participants to variables in the models is more than double the rule-of-thumb minimum of 10 per variable.^49^ Furthermore, the cross-sectional design precludes causal interpretations of the associations between variables, as mentioned. Indeed, the reverse associations between post-traumatic stress and pain outcomes are plausible, as highlighted within the mutual maintenance model,^17,50^ for example. Rather than identifying causal relationships, the current study should be interpreted as correlational and providing evidence only for the existence of associations between the variables. Finally, we dichotomized ethnicity for our analyses due to the relatively small numbers of participants within certain ethnic groups. While dichotomizing facilitated statistical analyses, doing so limits a more nuanced understanding of potential differences in the studied variables between specific groups.

In conclusion, exposure to trauma and the presence of clinically elevated PTSS are common among people engaging in ACT-based treatment for pain. Post-traumatic stress is associated with adverse pain-related outcomes. Trauma-informed practice is important when assessing suitability for and providing ACT-based treatment for pain. Research is needed to investigate the impact of trauma symptoms on treatment outcomes following ACT-based treatment for pain. Longitudinal and experimental intervention research are needed to disentangle the nature of the causal relationships between post-traumatic stress, psychological flexibility, and pain-related outcomes.

## Supplemental material

Supplemental Material - A cross-sectional study of the prevalence and correlates of post-traumatic stress among people attending acceptance and commitment therapy for chronic pain Supplemental Material for A cross-sectional study of the prevalence and correlates of post-traumatic stress among people attending acceptance and commitment therapy for chronic pain by Kezia Kadri, Cara Keating, Madeleine Anderson, David Novelli, Emily McBride, and Whitney Scott in British Journal of Pain

## Data Availability

Sharing of anonymized data for research purposes consistent with the original aims of the overall research database will be considered upon reasonable request.*

## References

[1] Jennifer S Brady BR Ibrahim MM , et al. Co-occurrence of chronic pain and anxiety/depression symptoms in US adults: prevalence, functional impacts, and opportunities. Pain 2024; 165: 666–673. 10.1097/j.pain.0000000000003056 PMC10859853 37733475

[2] Tidmarsh LV Harrison R Ravindran D , et al. The influence of adverse childhood experiences in pain management: mechanisms, processes, and trauma-informed care. Front Pain Res 2022; 3: 923866. 10.3389/fpain.2022.923866 PMC9226323 35756908

[3] American Psychiatric Association . Diagnostic and statistical manual of mental disorders. 5th ed. : American Psychiatric Association, 2013.

[4] Siqveland J Hussain A Lindstrøm JC , et al. Prevalence of posttraumatic stress disorder in persons with chronic pain: a meta-analysis. Front Psychiatry 2017; 8: 164. 10.3389/fpsyt.2017.00164 PMC5603802 28959216

[5] Kessler RC Berglund P Demler O , et al. Lifetime prevalence and age-of-onset distributions of DSM-IV disorders in the National Comorbidity Survey Replication. Arch Gen Psychiatry 2005; 62: 593–602. 10.1001/archpsyc.62.6.593 15939837

[6] Umucu E Reyes A Carrola P , et al. Pain intensity and mental health quality of life in veterans with mental illnesses: the intermediary role of physical health and the ability to participate in activities. Qual Life Res 2021; 30: 479–486. 10.1007/s11136-020-02642-y PMC7515555 32974882

[7] El Sount CR-O Windthorst P Denkinger J , et al. Chronic pain in refugees with posttraumatic stress disorder (PTSD): a systematic review on patients' characteristics and specific interventions. J Psychosom Res 2019; 118: 83–97. 10.1016/j.jpsychores.2018.07.014 30078503

[8] Åkerblom S Perrin S Rivano FM , et al. The impact of PTSD on functioning in patients seeking treatment for chronic pain and validation of the posttraumatic diagnostic scale. Int J Behav Med 2017; 24: 249–259. 10.1007/s12529-017-9641-8 PMC5344943 28194719

[9] Giummarra MJ Casey SL Devlin A , et al. Co-occurrence of posttraumatic stress symptoms, pain, and disability 12 months after traumatic injury. Pain Reports 2017; 2: e622. 10.1097/PR9.0000000000000622 PMC5777683 29392235

[10] Herbert MS Malaktaris AL Dochat C , et al. Acceptance and commitment therapy for chronic pain: does post-traumatic stress disorder influence treatment outcomes? Pain Med 2019; 20: 1728–1736. 10.1093/pm/pny272 30602034

[11] Hickling LM Allani S Cella M , et al. A systematic review with meta-analyses of the association between stigma and chronic pain outcomes. Pain 2024; 165: 1689–1701. 10.1097/j.pain.0000000000003243 PMC11247453 38776178

[12] Benfer N Howell MK Lucksted A , et al. Self-stigma and PTSD: conceptualization and implications for research and treatment. Psychiatr Serv 2023; 74: 1081–1083. 10.1176/appi.ps.20220397 36935625

[13] Manuel J Rudolph L Beissner F , et al. Traumatic events, posttraumatic stress disorder, and central sensitization in chronic pain patients of a German university outpatient pain clinic. Psychosom Med 2023; 85: 351–357. 10.1097/psy.0000000000001181 PMC10171308 36825929

[14] Linnemørken LTB Granan L-P Reme SE . Prevalence of posttraumatic stress symptoms and associated characteristics among patients with chronic pain conditions in a Norwegian University Hospital outpatient pain clinic. Front Psychol 2020; 11: 749. 10.3389/fpsyg.2020.00749 PMC7215085 32431641

[15] Williams ACC Fisher E Hearn L , et al. Psychological therapies for the management of chronic pain (excluding headache) in adults. Coch Database Syst Rev 2020; 8: CD007407. 10.1002/14651858.CD007407.pub4 PMC7437545 32794606

[16] National Institute for Health and Care Excellence . Guideline NG193. Chronic pain (primary and secondary) in over 16s: assessment of all chronic pain and management of chronic primary pain. https://www.nice.org.uk/guidance/ng193 (2021, Accessed 1 September 2025). 33939353

[17] Asmundson GJ Coons MJ Taylor S , et al. PTSD and the experience of pain: research and clinical implications of shared vulnerability and mutual maintenance models. Can J Psychiatry 2002; 47: 930–937. 10.1177/070674370204701004 12553128

[18] Åkerblom S Perrin S Fischer MR , et al. The relationship between posttraumatic stress disorder and chronic pain in people seeking treatment for chronic pain: the mediating role of psychological flexibility. Clin J Pain 2018; 34: 487–496. 10.1097/ajp.0000000000000561 29016388

[19] Hayes SC Levin ME Plumb-Vilardaga J , et al. Acceptance and Commitment Therapy and contextual behavioral science: examining the progress of a distinctive model of behavioral and cognitive therapy. Behav Ther 2013; 44: 180–198. 10.1016/j.beth.2009.08.002 PMC3635495 23611068

[20] McCracken LM . Psychological flexibility, chronic pain, and health. Ann Rev Psychol 2024; 75: 601–624. 10.1146/annurev-psych-020223-124335 37585667

[21] McCracken LM Yu L Vowles KE . New generation psychological treatments in chronic pain. BMJ 2022; 376: e057212. 10.1136/bmj-2021-057212 35228207

[22] Hughes LS Clark J Colclough JA , et al. Acceptance and commitment therapy (ACT) for chronic pain: a systematic review and meta-analyses. Clin J Pain 2017; 33: 552–568. 10.1097/AJP.0000000000000425 27479642

[23] Duncan P Murphy M Man M-S , et al. Development and validation of the multimorbidity treatment burden questionnaire (MTBQ). BMJ Open 2020; 8: e019413. 10.1136/bmjopen-2017-019413 PMC5900423 29654011

[24] Weathers FW Litz BT Keane TM , et al. The PTSD checklist for DSM-5 (PCL-5). https://www.ptsd.va.gov/professional/assessment/documents/PCL5_Standard_form.pdf (2013, accessed 2 April 2025).

[25] Wortmann JH Jordan AH Weathers FW , et al. Psychometric analysis of the PTSD Checklist-5 (PCL-5) among treatment-seeking military service members. Psychol Assess 2016; 28: 1392–1403. 10.1037/pas0000260 26751087

[26] Dworkin RH Turk DC Farrar JT , et al. Core outcome measures for chronic pain clinical trials: IMMPACT recommendations. Pain 2005; 113: 9–19. 10.1016/j.pain.2004.09.012 15621359

[27] Atisook R Euasobhon P Saengsanon A , et al. Validity and utility of four pain intensity measures for use in international research. J Pain Res 2021; 14: 1129–1139. 10.2147/jpr.s303305 PMC8071079 33907460

[28] Cleeland CS Ryan K . Pain assessment: global use of the Brief pain Inventory. Ann Acad. Med 1994; 23: 129–138. 8080219

[29] Keller S Bann CM Dodd SL , et al. Validity of the Brief Pain Inventory for use in documenting the outcomes of patients with noncancer pain. Clinical J Pain 2004; 20: 309–318. 10.1097/00002508-200409000-00005 15322437

[30] Mundt JC Marks IM Shear MK , et al. The Work and Social Adjustment Scale: a simple measure of impairment in functioning. Brit J Psychiatry 2002; 180: 461–464. 10.1192/bjp.180.5.461 11983645

[31] Zahra D Qureshi A Henley W , et al. The Work and Social Adjustment Scale: reliability, sensitivity and value. Int J Psychiatry Clin Pract 2014; 18: 131–138. 10.3109/13651501.2014.894072 24527886

[32] Kroenke K Spitzer RL Williams JB . The PHQ-9: validity of a brief depression severity measure. J Gen Internal Med 2001; 16: 606–613. 10.1046/j.1525-1497.2001.016009606.x PMC1495268 11556941

[33] Rolffs JL Rogge RD Wilson KG . Disentangling components of flexibility via the hexaflex model: development and validation of the Multidimensional Psychological Flexibility Inventory (MPFI). Assessment 2018; 25: 458–482. 10.1177/1073191116645905 27152011

[34] Sundström FT Lavefjord A Buhrman M , et al. Assessing psychological flexibility and inflexibility in chronic pain using the multidimensional psychological flexibility inventory (MPFI). J Pain 2023; 24: 770–781. 10.1016/j.jpain.2022.11.010 36442815

[35] Cervantes-Perea W Martínez-Bacaicoa J Gámez-Guadix M . Psychological flexibility and inflexibility of university students: an in-depth qualitative study. Int J Environ Res Public Health 2025; 22: 1141. 10.3390/ijerph22071141 PMC12294234 40724207

[36] Molina Y Choi SW Cella D , et al. The Stigma Scale for Chronic Illnesses 8-item version (SSCI-8): development, validation and use across neurological conditions. Int J Behav Med 2013; 20: 450–460. 10.1007/s12529-012-9243-4 PMC3758464 22639392

[37] Scott W Yu L Patel S , et al. Measuring stigma in chronic pain: preliminary investigation of instrument psychometrics, correlates, and magnitude of change in a prospective cohort attending interdisciplinary treatment. J Pain 2019; 20: 1164–1175. 10.1016/j.jpain.2019.03.011 30940501

[38] Green CR Ndao-Brumblay SK Nagrant AM , et al. Race, age, and gender influences among clusters of African American and white patients with chronic pain. J Pain 2004; 5: 171–182. 10.1016/j.jpain.2004.02.227 15106130

[39] Elmose Andersen T Hansen M Lykkegaard RS , et al. The association of probable PTSD at baseline and pain-related outcomes after chronic pain rehabilitation: a comparison of DSM-5 and ICD-11 criteria for PTSD. Eur J Pain 2022; 26: 709–718. 10.1002/ejp.1899 34939270

[40] Jones MWH Cook N McGrath BM , et al. Examining PTSD prevalence among underserved populations in the integrated community mental health setting 2019 to 2022. J Prim Care Community Health 2024; 15: 21501319241291751. 10.1177/21501319241291751 PMC11555738 39526841

[41] Buscemi V Chicken J Mahy T , et al. An updated audit of the patient selection process for pain management programmes in a specialty care service before and during the COVID-19 pandemic. Brit J Pain 2023; 17: 226–238. 10.1177/20494637221147200 PMC9791070 37337591

[42] Åkerblom S Perrin S Fischer MR , et al. Treatment outcomes in group-based cognitive behavioural therapy for chronic pain: an examination of PTSD symptoms. Eur J Pain 2020; 24: 807–817. 10.1002/ejp.1530 31904136

[43] Stander VA Thomsen CJ Highfill-McRoy RM . Etiology of depression comorbidity in combat-related PTSD: a review of the literature. Clin Psychol Review 2014; 34: 87–98. 10.1016/j.cpr.2013.12.002 24486520

[44] Leeuw M Goossens ME Linton SJ , et al. The fear-avoidance model of musculoskeletal pain: current state of scientific evidence. J Behav Med 2007; 30: 77–94. 10.1007/s10865-006-9085-0 17180640

[45] Montag LT Salomons TV Wilson R , et al. Examining the roles of depression, pain catastrophizing, and self-efficacy in quality of life changes following chronic pain treatment. Can J Pain 2023; 7: 2156330. 10.1080/24740527.2022.2156330 PMC9980521 36874232

[46] UK Government . Guidance: working definition of trauma-informed practice. Report, Office for Health Improvement and Disparities, UK. https://www.gov.uk/government/publications/working-definition-of-trauma-informed-practice/working-definition-of-trauma-informed-practice (2022, accessed 3 September 2025).

[47] Onwumere J Stubbs B Stirling M , et al. Pain management in people with severe mental illness: an agenda for progress. Pain 2022; 163: 1653–1660. 10.1097/j.pain.0000000000002633 PMC9393797 35297819

[48] Lewis C Roberts NP Andrew M , et al. Psychological therapies for post-traumatic stress disorder in adults: systematic review and meta-analysis. Eur J Psychotraumatol 2020; 11: 1729633. 10.1080/20008198.2020.1729633 PMC7144187 32284821

[49] VanVoorhis CW Morgan BL . Understanding power and rules of thumb for determining sample sizes. Tutor Quant Methods Psychol 2007; 3: 43–50.

[50] Liedl A O'Donnell M Creamer M , et al. Support for the mutual maintenance of pain and post-traumatic stress disorder symptoms. Psychol Med 2010; 40: 1215–1223. 10.1017/S0033291709991310 19811699

