# Supplementary material for: A cross-sectional study of the prevalence and correlates of post-traumatic stress among people attending acceptance and commitment therapy for chronic pain
Source: Br J Pain. 2026 Oct 16:20494637261497966. Online ahead of print. doi: 10.1177/20494637261497966 (PMC13675411; doi:10.1177/20494637261497966)
Supplement: Supplementary file 1 [file sj-pdf-1-bjp-10.1177_20494637261497966.pdf]

Supplementary Materials

Supplementary Table 1
Regressions examining each PCL-5 symptom cluster severity in relation to depression

	Independent Variable	F change	Df	*P*	R^2_{adj}	ΔR^2_{adj}	β	Unstandardised B (SE)	*P*
				Model 1 – PCL-5 Cluster B as Independent Variable					
Step 1	Age	1.86	(5, 172)	.103	.02		-.06	-0.02 (0.03)	.468
	Gender						-.06	-0.90 (1.20)	.455
	Ethnicity						-.07	-0.84 (0.92)	.359
	Pain location						-.19	-2.32 (0.94)	.015
	Working status						.07	0.78 (0.85)	.357
Step 2	Pain intensity	18.79	(1, 171)	<.001	.12	.10	.31	1.18 (0.27)	<.001
Step 3	PCL-5 Cluster B	50.89	(1, 170)	<.001	.32	.20	.46	0.41 (0.06)	<.001
				Model 2 – PCL-5 Cluster C as Independent Variable					
Step 1	Age	1.88	(5, 167)	.100	.03		-.06	-0.02 (0.03)	.474
	Gender						-.05	-0.82 (1.20)	.495
	Ethnicity						-.05	-0.64 (0.94)	.494
	Pain location						-.20	-2.42 (0.95)	.012
	Work status						.07	0.82 (0.87)	.345
Step 2	Pain intensity	25.46	(1, 166)	<.001	.15	.12	.36	1.38 (0.27)	<.001
Step 3	PCL-5 Cluster C	38.34	(1, 166)	<.001	.31	.19	.41	0.83 (0.13)	<.001
				Model 3 - PCL-5 Cluster D as Independent Variable					
Step 1	Age	1.87	5, 173	.101	.02		-.05	-0.02 (0.03)	.530
	Gender						-.06	-0.88 (1.20)	.465
	Ethnicity						-.08	-0.91 (0.92)	.320
	Pain location						-.19	-2.39 (0.94)	.012
	Work status						.06	0.69 (0.85)	.414
Step 2	Pain intensity	19.92	1, 172	<.001	.12	.10	.32	1.20 (0.27)	<.001
Step 3	PCL-5 Cluster D	109.72	1, 171	<.001	.46	.34	.60	0.46 (0.04)	<.001
				Model 4 - PCL-5 Cluster E as Independent Variable					
Step 1	Age	1.85	(5, 172)	.105	.02		-.05	-0.02 (0.03)	.500
	Gender						-.06	-0.89 (1.20)	.458
	Ethnicity						-.07	-0.86 (0.92)	.348
	Pain location						-.19	-2.35 (0.94)	.014
	Work status						.07	0.76 (0.85)	.374
Step 2	Pain intensity	20.08	(1, 171)	<.001	.12	.10	.32	1.21 (0.27)	<.001
Step 3	PCL-5 Cluster E	65.65	(1, 170)	<.001	.36	.24	.50	0.49 (0.06)	<.001

Note. All Standardised β were from final regression models. Gender, ethnicity, pain location, and work status were dichotomized, with the reference categories being male (gender), white (ethnicity), pain location not all over (pain location), employed, including part time and student (work status), respectively.
Df = degrees of freedom, ΔR^2adj = change in R^2, SE = standard error.

Supplementary Table 2

Linear regressions using categorical trauma variable in relation to pain outcomes

	Independent Variable	F change	Df	*p*	R^2_{adj}	ΔR^2_{adj}	β	Unstandardised B (SE)	*p*
				Model 1 – Dependent: Pain intensity					
Step 1	Age	3.18	(4, 377)	.014	.02		.11	0.01 (.01)	.038
	Gender						.01	0.06 (.21)	.792
	Ethnicity						.05	0.17 (.17)	.314
	Pain location						-.14	-0.46 (.17)	.007
Step 2	Probable PTSD	.10	(1, 376)	.752	.02	.00	.02	0.05 (.17)	.752
				Model 2 – Dependent: Pain interference					
Step 1	Age	2.40	(5, 373)	.037	.02		-.06	-0.01 (.01)	.177
	Gender						-.01	-0.06 (.19)	.771
	Ethnicity						-.05	-0.16 (.15)	.299
	Pain location						-.05	-0.20 (.16)	.218
	Work status						.07	0.23 (.15)	.116
Step 2	Pain intensity	158.47	(1, 372)	<.001	.31	.29	.55	0.61 (.05)	<.001
Step 3	Probable PTSD	13.41	(1, 371)	<.001	.33	.02	.16	0.57 (.16)	<.001
				Model 3 - Dependent: Impact on functioning					
Step 1	Age	7.35	(5, 372)	<.001	.08		.01	0.01 (.03)	.787
	Gender						-.01	-0.21 (.90)	.809
	Ethnicity						-.06	-0.94 (.71)	.188
	Pain location						-.11	-1.85 (.74)	.013
	Work status						.20	3.04 (.68)	<.001
Step 2	Pain intensity	67.62	(1, 371)	<.001	.22	.14	.38	1.86 (.22)	<.001
Step 3	Probable PTSD	19.88	(1, 370)	<.001	.26	.04	.20	3.24 (.73)	<.001
				Model 4 - Dependent: Depression					
Step 1	Age	7.35	(5, 371)	<.001	.08		-.12	-0.05 (.02)	.008
	Gender						-.02	-0.28 (.71)	.696
	Ethnicity						-.08	-0.95 (.56)	.090
	Pain location						-.19	-2.43 (.58)	<.001
	Work status						.09	1.04 (.53)	.051
Step 2	Pain intensity	26.38	(1, 370)	<.001	.14	.06	.25	0.94 (.17)	<.001
Step 3	Probable PTSD	53.29	(1, 369)	<.001	.24	.10	.33	4.18 (.57)	<.001

Note. All Standardised β were from final regression models. Gender, ethnicity, pain location, work status and PCL-5 Probable PTSD were dichotomized, with the reference categories being male (gender), white (ethnicity), pain location not all over (pain location), employed, including part time and student (work status), and no exposure or subthreshold PTSS (PCL-5 <31;PCL-5 Probable PTSD), respectively.
Df = degrees of freedom, ΔR^2adj = change in R^2, SE = standard error.

Supplementary Table 3

Regressions including categorical trauma variable and psychological flexibility as factors associated with pain outcomes

	Independent Variable	F change	Df	*p*	R^2_{adj}	ΔR^2_{adj}	β	Unstandardised B (SE)	*p*
				Model 1 – Dependent: Pain intensity					
Step 1	Age	3.21	4, 358	.013	.02		.11	.01 (.01)	.050
	Gender						-.01	-.03 (.22)	.898
	Ethnicity						.06	.19 (.17)	.284
	Pain location						-.15	-.48 (.18)	.007
Step 2	Psychological flexibility	.002	1, 357	.961	.02	.00	.01	.01 (.09)	.922
Step 3	Probable PTSD	.736	1, 356	.736	.02	.00	.02	.06 (.02)	.736
				Model 2 – Dependent: Pain interference					
Step 1	Age	2.71	5, 354	.020	.02		-.04	-.01 (.01)	.383
	Gender						-.01	-.06 (.20)	.770
	Ethnicity						-.03	-.12 (.16)	.442
	Pain location						-.08	-.27 (.16)	.089
	Work Status						.06	.21 (.15)	.155
Step 2	Pain intensity	151.46	1, 353	<.001	.32	.20	.55	.60 (.05)	<.001
Step 3	Psychological flexibility	12.85	1, 352	<.001	.34	.02	-.14	-.25 (.08)	.002
Step 4	Probable PTSD	10.75	1, 351	.001	.36	.02	.14	.53 (.16)	.001
				Model 3 - Dependent: Impact on functioning					
Step 1	Age	6.91	5, 355	<.001	.08		.03	.01 (.03)	.587
	Gender						.00	.06 (.94)	.948
	Ethnicity						-.06	-.91 (.75)	.228
	Pain location						-.13	-2.01 (.77)	.008
	Work status						.19	2.89 (.70)	<.001
Step 2	Pain intensity	64.94	1, 354	<.001	.22	.14	.38	1.88 (.23)	<.001
Step 3	Psychological flexibility	8.15	1, 353	.005	.23	.01	-.11	-.89 (.39)	.023
Step 4	Probable PTSD	15.97	1, 352	<.001	.26	.03	.18	3.07 (.77)	<.001
				Model 4 - Dependent: Depression					
Step 1	Age	7.71	3, 353	<.001	.09		-.08	-.04 (.02)	.073
	Gender						.01	.08 (.70)	.914
	Ethnicity						-.05	-.55 (.56)	.321
	Pain location						-.21	-2.70 (.57)	<.001
	Work status						.07	.83 (.52)	.113
Step 2	Pain intensity	25.89	1, 352	<.001	.15	.06	.25	.96 (.17)	<.001

Step 3	Psychological flexibility	51.91	1, 351	<.001	.25	.10	-.30	-1.89 (.29)	<.001
Step 4	Probable PTSD	42.01	1, 350	<.001	.33	.08	.29	3.69 (.57)	<.001

Note. All Standardised β were from final regression models. Gender, ethnicity, pain location, and work status were dichotomized. Reference categories for dichotomized variables were male (gender), white (ethnicity), pain location not all over (pain location), employed including part time and student (work status), and no exposure or subthreshold PTSS (PCL-5 <31;PCL-5 Probable PTSD), respectively.
Df = degrees of freedom, ΔR^2adj = change in R^2, SE = standard error.

Supplementary Table 4
Regressions including categorical trauma variable and psychological inflexibility as factors associated with pain outcomes

	Independent Variable	F change	Df	*p*	R^2_{adj}	ΔR^2_{adj}	β	Unstandardised B (SE)	*p*
				Model 1 – Dependent: Pain intensity					
Step 1	Age	2.94	4, 361	.021	.02		.12	.01 (.01)	.031
	Gender						-.01	-.03 (.22)	.878
	Ethnicity						.06	.20 (.17)	.246
	Pain location						-.13	-.43 (.18)	.014
Step 2	Psychological inflexibility	1.29	1, 360	.257	.02	.00	.06	.10 (.10)	.289
Step 3	Probable PTSD	.00	1, 359	.975	.02	.00	.00	.01 (.19)	.975
				Model 2 – Dependent: Pain interference					
Step 1	Age	2.27	5, 357	.048	.02		-.04	-.01 (.01)	.320
	Gender						-.03	-.14 (.20)	.486
	Ethnicity						-.02	-.07 (.16)	.633
	Pain location						-.05	-.19 (.16)	.226
	Work status						.06	.21 (.15)	.147
Step 2	Pain intensity	162.64	1, 356	<.001	.32	.30	.55	.61 (.05)	<.001
Step 3	Psychological inflexibility	15.47	1, 355	<.001	.35	.03	.13	.25 (.09)	.005
Step 4	Probable PTSD	7.64	1, 354	.006	.36	.01	.12	.46 (.17)	.006
				Model 3 - Dependent: Impact on functioning					
Step 1	Age	6.90	5, 355	<.001	.08		-.05	-.02 (.02)	.247
	Gender						-.06	-.88 (.64)	.169
	Ethnicity						-.02	-.23 (.51)	.654
	Pain location						-.17	-2.12 (.52)	<.001
	Work status						.06	.75 (.48)	.119
Step 2	Pain intensity	27.26	1, 354	<.001	.14	.06	.23	.87 (.15)	<.001
Step 3	Psychological inflexibility	153.46	1, 353	<.001	.40	.26	.47	3.05 (.29)	<.001

Step 4	Probable PTSD	18.13	1, 352	<.001	.43	.03	.18	2.32 (.54)	<.001
Model 4 - Variable: Depression									
Step 1	Age	7.41	5, 358	<.001	.08		.03	.02 (.03)	.486
	Gender						-.02	-.31 (.90)	.734
	Ethnicity						.03	-.51 (.72)	.479
	Pain location						-.11	-1.77 (.73)	.016
	Work status						.21	3.13 (.67)	<.001
Step 2	Pain intensity	73.19	1, 357	<.001	.24	.16	.39	1.90 (.22)	<.001
Step 3	Psychological inflexibility	19.43	1, 356	<.001	.27	.03	.14	1.22 (.41)	.003
Step 4	Probable PTSD	14.36	1, 355	<.001	.30	.03	.18	2.91 (.77)	<.001

Note. All Standardised β were from final regression models. Gender, ethnicity, pain location, and work status were dichotomized. Reference categories for dichotomized variables were male (gender), white (ethnicity), pain location not all over (pain location), employed including part time and student (work status), and no exposure or subthreshold PTSS (PCL-5 <31;PCL-5 Probable PTSD), respectively.
Df = degrees of freedom, ΔR^2adj = change in R^2, SE = standard error.
